# Supplementary material for: Non-linear enhancement of ultrafast X-ray diffraction through transient resonances
Source: Nat Commun. 2025 Jan 20;16:847. doi: 10.1038/s41467-025-56046-y (PMC11747624; doi:10.1038/s41467-025-56046-y)
Supplement: Supplementary file 1 — Supplementary Information [file 41467_2025_56046_MOESM1_ESM.pdf]

Supplements to Non-linear enhancement of ultrafast X-ray diffraction through transient resonances

Stephan Kuschel[1,2,3,4*], Phay J. Ho[5 *], Andre Al Haddad[5,6], Felix F. Zimmermann[1,2,7], Leonie Flueckiger[8], Matthew R. Ware[1,2], Joseph Duris[2], James P. MacArthur[2], Alberto Lutman[2], Ming-Fu Lin[2], Xiang Li[2,9], Kazutaka Nakahara[2], Jeff W. Aldrich[2], Peter Walter[2], Linda Young[5,10], Christoph Bostedt[5,6,11], Agostino Marinelli[2*], and Tais Gorkhover[1,2,3*]

[1]Stanford PULSE Institute, SLAC National Accelerator Laboratory, 2575 Sand Hill Rd, Menlo Park, CA 94025, USA
[2]SLAC National Accelerator Laboratory, 2575 Sand Hill Rd, Menlo Park, CA 94025, USA
[3]University of Hamburg, Institute for Experimental Physics/CFEL, Luruper Chaussee 149 22761 Hamburg, Germany
[4]Technical University Darmstadt, Institute of nuclear physics, Schlossgartenstr. 9, 64289 Darmstadt, Germany
[5]Chemical Sciences and Engineering Division, Argonne National Laboratory, 9700 S. Cass Avenue, Lemont, IL 60439, USA
[6]Paul Scherrer Institute, 5232 Villigen, Switzerland
[7]IOAP, TU Berlin, Hardenbergstrasse 36, 10623 Berlin
[8]La Trobe University, 1300 La Trobe, Australia
[9]J.R. Macdonald Laboratory, Department of Physics, Kansas State University, Manhattan, Kansas 66506, USA
[10]Department of Physics and James Franck Institute, The University of Chicago, Chicago, IL 60637, USA
[11]LUXS Laboratory for Ultrafast X-ray Sciences, Institute of Chemical Sciences and Engineering, École Polytechnique Fédérale de Lausanne (EPFL), CH-1015 Lausanne, Switzerland
[*]stephan.kuschel@tu-darmstadt.de
[*]tais.gorkhover@cfel.de
[*]pho@anl.gov
[*]marinelli@slac.stanford.edu

Supplementary Note 1. Methods simulations

We employed Monte-Carlo/Molecular-Dynamics (MC/MD) calculations to simulate the scattering cross sections of the Xe clusters [1–3] to model the full electron and nuclear dynamics in an atomistic manner during the full duration of the X-ray pulse. In more detail, the interaction of the atom with incident XFEL pulse is treated quantum mechanically with a Monte Carlo method by tracking explicitly the time-dependent quantum transition probability between different electronic configurations. The total transition rate, Γ, between different electronic configurations I and J is given by

$$\Gamma_{I,J} = \Gamma^{P}_{I,J} + \Gamma^{A}_{I,J} + \Gamma^{F}_{I,J} + \Gamma^{RE}_{I,J} + \Gamma^{EI}_{I,J} + \Gamma^{RC}_{I,J}. \tag{1}$$

Starting from the ground state of the neutral atom, we include the contribution from photoionization $\Gamma^{P}_{I,J}$, Auger decay $\Gamma^{A}_{I,J}$, fluorescence $\Gamma^{F}_{I,J}$, resonant excitation $\Gamma^{RE}_{I,J}$, electron-impact ionization $\Gamma^{EI}_{I,J}$ and electron-ion recombination $\Gamma^{RC}_{I,J}$. The cross sections and rates are calculated with Hartree-Fock-Slater model [2] with relativistic corrections and spin-orbit coupling in orbital energies, following the procedure outlined in reference [4]. Additionally, a molecular dynamics (MD) algorithm is used to propagate all particle trajectories (atoms/ions/electrons) forward in time in 1 attosecond steps. The cluster dynamics includes electromagnetic forces between the charged particles and van der waal forces among the neutral atoms.

The importance of understanding transient dynamics is that the incoming photons arriving at different times will scatter off the instantaneously populated transient states. Similar to the treatment presented in [3], the observed scattering response is characterized as a sum of the instantaneous scattering patterns weighted by the pulse fluence, $j_X(\tau,t)$, with FWHM duration τ and convolved with a Gaussian bandwidth profile, $g(\omega,\omega_x)$, with a central photon energy of ω_x, such that

$$\frac{d\sigma}{d\Omega}(\vec{q}) = \frac{d\sigma_{\text{th}}}{d\Omega}\frac{1}{\mathscr{F}}\int_0^{+\infty} d\omega \int_{-\infty}^{+\infty} dt\, g(\omega,\omega_x)\, j_X(\tau,t)\, |F_c(\vec{q},t)|^2, \tag{2}$$

where $d\sigma_{\text{th}}/d\Omega$ is the Thomson scattering cross section.

$$\mathscr{F} = \int_0^{+\infty} d\omega \int_{-\infty}^{+\infty} dt\, j_X(\tau,t) g(\omega,\omega_x) \tag{3}$$

is the fluence of an XFEL pulse, and $\int_0^{+\infty} d\omega g(\omega,\omega_x) = 1$. Here $F_c(\vec{q},t)$ is the time-dependent form factor of the target cluster and is given by

$$F_c(\vec{q},t) = \sum_{j=1}^{N_a} f_j(\vec{q},C_j(t)) e^{i\vec{q}\cdot\vec{R}_j(t)} + \sum_{j=1}^{N_e(t)} e^{i\vec{q}\cdot\vec{r}_j(t)}\,, \tag{4}$$

where N_a is the total number of atoms/ions, $\vec{R}_j(t)$, $C_j(t)$ and $f_j(\vec{q},C_j(t))$ are the position, the electronic configuration and the atomic form factor of the j-th atom/ion respectively. $N_e(t)$ is the number of delocalized electrons within the focal region of the X-ray pulse and $\vec{r}_j(t)$ are their positions.

To simulate the scattering cross section of atomic xenon, we used the Monte-Carlo rate equation [5]. We explicitly track the time-dependent quantum transition probability between different electronic configurations in atoms exposed to X-ray pulse. The total transition rate, Γ, between different electronic configurations I and J is same as that used in MC/MD calculation, except $\Gamma_{I,J}^{EI}$ and $\Gamma_{I,J}^{RC}$ are set to zero.

The total scattering cross section of j-th atom with an electronic configuration of C_j is computed as

$$\sigma = \int d\Omega \frac{d\sigma_{\text{th}}}{d\Omega} |f_j(\vec{q})|^2 \tag{5}$$

where $d\sigma_{\text{th}}/d\Omega$ is the Thomson scattering cross section and the form factor

$$f_j(\vec{q}) = f_{0,j}(\vec{q},C_j) + f_j'(\omega) + i f_j''(\omega) \tag{6}$$

where $f_{0,j}(\vec{q},C_j)$ is the Fourier transform of the electron density and $\vec{q}$ is the momentum transfer vector. f' and f'' are the real and imaginary parts of the anomalous (resonant) scattering terms, and they are related to Kramers-Kronig relation. For each electronic configuration, f'' is computed using the optical theorem,

$$f''(\omega) = \sum_v \frac{\omega}{4\pi\alpha} \sigma_v^{(\text{PI})} + \sum_{u,v} \frac{\pi}{2} \omega_{vu} f_{vu}^{(\text{osc})} \frac{1}{\pi} \frac{\gamma_{vu}/2}{(\omega-\omega_{vu})^2 + \gamma_{vu}^2/4} \tag{7}$$

where $\sigma_v^{(\text{PI})}$ is the photoionization cross section of the occupied u-subshell, ω_{vu} is the transition energy of electron in the occupied v-subshell to the u-subshell with a natural linewidth of γ_{vu}, and $f_{vu}^{(\text{osc})}$ is the associated oscillator strength of this transition and is connected with the overlap between the v-th and u-th states.

Supplementary Note 2. Simulations on cluster expansion

At the beginning of the simulation, neutral atoms interact through a Lennard-Jones potential. X-ray photoionisation and Auger processes produce ions and free electrons, adding Coulombic forces to the cluster. These electrons in turn trigger collisional ionization which rapidly becomes the dominant ionization process. The delocalized but trapped electrons heat up the cluster from the inside. This process peaks around -100 fs together with the acceleration of the Xenon ions as seen in Supplementary Figure 1. In turn the cluster expands well before the maximum of the XFEL pulse arrives. The simulation results in Supplementary Figure 5 (solid lines only) are based on the same method and additionally integrated the scattering cross section over all xenon atoms and the entire exposure time.

Supplementary Note 3.Experimental Setup

A schematic of our experiment is exhibited in Supplementary Figure 2: Individual Xe nanoparticles with diameters 40-150 nanometers intersect the path of the focused and intense single X-ray FEL pulses at the LAMP endstation

at the Linac Coherent Light Source (LCLS) [6]. Single particle, single exposure snapshots were recorded using a p-n junction charge coupled devices (pnCCDs)[7] located at two different positions further downstream from the interaction region. The particle size is encoded with ± 0.3 nm precision into the Airy pattern-like diffraction (see supplement, section Cluster size fitting model). Single-exposure radial diffraction profiles collected by the rear and front detectors are in agreement with theoretical plots for X-ray diffraction by a perfect sphere within Born approximation [8]. The deviations from a perfect sphere can be mostly attributed to sharp facets near the surface.

The experiment was conducted at the AMO hutch of the LCLS light source at SLAC National Laboratory in the US. Xenon nanoclusters were injected into the focal region of the X-ray beam (see Supplementary Figure 2). A pnCCD detector was positioned 130 mm downstream of the interaction point thus called the "front" pnCCD Another, the "rear" pnCCD, was positioned at a distance of 732 mm downstream the interaction point. The Xe cluster density was adjusted to record diffraction patterns of single Xe nanoclusters only. As those clusters are almost perfect spheres their size can be reconstructed with high precision. The number of scattered photons on the detector and the exact size of the cluster combined allow to calculate the scattering cross section of a single Xe atom. See (see supplement, section Cluster size fitting model) for details. This step is of crucial importance for the analysis as it allows us to correct the scattering for the cluster size in each XFEL pulse and thus calculate the scattering cross section.

The XFEL focal spot of 1.5 μm (FWHM) is over one order of magnitude wider than the nanoparticles and thus, each nanoparticle is exposed to a different but uniform power density. Based on statistical considerations one can assume that brightest 0.1 % of all shots were recorded close to the FEL focus center. The absolute exposure fluence in the focus center has been calibrated using fluorescence yield per atom of the most intense shot. We chose brightest shots from the 5 fs measurement at 730 eV for absolute fluence calibration. Here, the highest fluorescence yield indicates that only 0.3 photons per atom were absorbed (see Supplementary Figure 5, lower left and right graphs). In this condition, the linear semi-classical model of diffraction is still valid and the corresponding FEL fluence can be estimated. For other data points, the FEL fluence was linearly scaled to the 5 fs measurement value of the gas detector. The gas detector measures an independent value for the FEL pulse energy which varied for different photon energies.

The obtained value for FEL fluence was cross checked with experimental settings such as beamline transmission, focus size, quantum efficiency of the detectors and also through a independent Ar ionization spectrum measurement. The temporal profiles of 5-200 fs FEL pulses are not precisely known, the pulse duration is based on indirect electron bunch measurements.

We systematically scanned the photon energy around the Xenon M-shell absorption edge ($\sim$700 eV). Three different pulse durations were used throughout the experiment:
1) 200 fs duration,

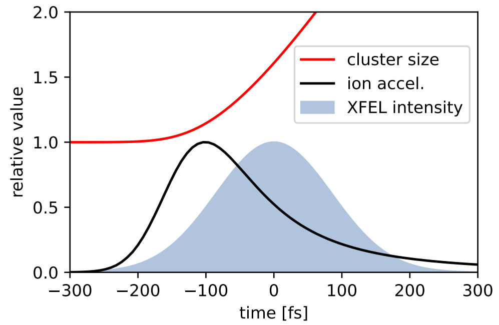

Supplementary Figure 1. Simulation of Xe cluster expansion due to XFEL irradiation with a 200 fs pulse: The cluster's size is measured by the standard deviation of all particle positions within the simulation and the "ion acceleration" is its second derivative. The acceleration peaks around -100 fs, where the ionization rate also has a maximum due to collisional ionization (not shown).

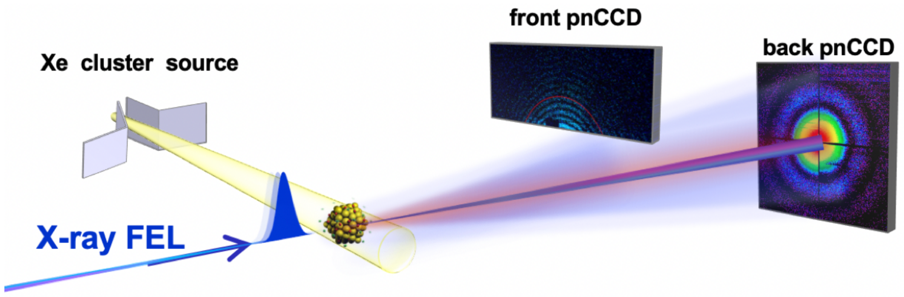

Supplementary Figure 2. Schematic of the experimental setup at the AMO Hutch at LCLS: Intense X-ray FEL pulses were focused and intersected with a stream of Xe clusters. The resulting single- shot single-particle X-ray diffraction patterns were recorded using pnCCD detectors positioned further downstream of the interaction point. The front pnCCD is positioned in a distance of 130 mm and the rear pnCCD at 732 mm from the X-ray focus. The outer regions of the front pnCCD, where the diffraction signal has vanished still register single fluorescence photons.

name	color	τ [fs]	Energy [mJ]	Fluence [μJ/μm^2]	Photon Energy
XLEAP	orange	<1	0.03	0.2 ± 0.1	Xe M-edge
200fsfull	dark blue	200	1.5	12 ± 2	Xe M-edge
200fsatt	purple	200	0.02	0.16 ± 0.02	Xe M-edge
5fs	light blue	5	0.01	0.08 ± 0.03	Xe M-edge
1500 eV	red	5	0.1	1.6 ± 0.3	1500 eV

Supplementary Table 1. Summarized experimental conditions with color coding used throughout the publication. The fluence is shown in average values over each dataset and $\pm$ values indicate the stability at a single photon energy. The underlying data is the pulse energy measured by the gas detectors.

2) 5 fs pulse duration by inserting a slotted foil[9] and
3) the recently developed XLEAP[10] mode delivering sub-fs pulses.
For each setting, the attenuation was adjusted to keep the photon number constant in each X-ray pulse duration within the precision of the attenuator, all average parameters are summarized in Supplementary Table 1 Inside the high-vacuum chamber the X-ray beam was focused down to a size of $\sim 1.5\mu m$ (FWHM) in diameter using the existing KB-mirrors of the AMO endstation. The beamline transmission was measured to be 0.2 around 700 eV and 0.4 around 1500 eV.

The Xe clusters were formed by supersonic expansion of xenon at 9 bar backing pressurethrough a 200 μm conical nozzle with a half-opening angle of 4° cooled to 245 K. The pulsed cluster jet was skimmed twice. An additional piezo-driven slit skimmer right before the interaction region was adjusted such that, on average, less than one particle was in the FEL focus.

Supplementary Note 4. Cluster size fitting model

We analyzed single diffraction snapshots of individual clusters and fitted the corresponding diffraction radial profile depending on the cluster size. As clusters are mostly spherical, we choose the analytical diffraction pattern of a hard sphere as our working model [8]. The adjustable parameters of the aforementioned fit determine the particle size (namely its radius R) and the brightness of the recorded diffraction image encoded in the form of the parameter I_0. We extracted the absolute scattering cross section from a single image by combining both fit parameters with the calibrated X-ray exposure fluence values (last section, Supplements).

The one-dimensional plot of the X-ray diffraction patterns from a solid sphere is given by and displayed in

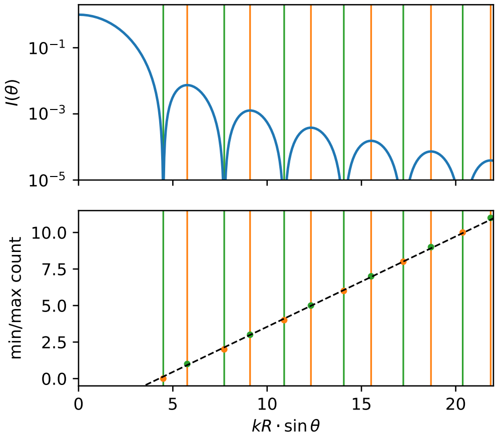

Supplementary Figure 3. The diffraction pattern of a solid sphere $I(\theta)$ is shown on the top. The green (orange) lines mark the minima (maxima) of the this function. The fact that maxima and minima are almost equally spaced is used to estimate their spacing and thus estimate the particle radius R. This estimated value is then used as a starting value for fitting the analytical diffraction pattern $I(\theta)$ to the measured one and thus determine R as well as I_0. This is performed separately for every measured single shot diffraction pattern in the experiment.

Supplementary Figure 3 (top, blue line):

$$I(\theta) = I_0 \cdot \frac{9}{x^2} \left(\frac{\sin(x)}{x^2} - \frac{\cos(x)}{x} \right)^2 \tag{8}$$

with $x = kR \sin\theta = \frac{2\pi}{\lambda} \cdot R \cdot \sin\theta$ and the sphere's radius R. The fitting route starts with a reasonable estimate for I_0 and R. This is particularly important for the parameter R, as the fitting routine may otherwise converge to a local minimum. For the numerical calculations, it is much more practical to write $x = kR\sin\theta$ as $x = 4.493 \cdot \frac{x_{\text{pxl}}}{x_1}$ where x_{pxl} is the x-value in pixel on the detector and x_1 is the pixel number of the first minimum of the function $I(\theta)$. The number 4.493 is the position of the first minimum of $I(\theta)$ as seen in Supplementary Figure 3 and was determined numerically.

Any given individually measured single shot diffraction image is first preprocessed by masking invalid detector regions and over-exposed pixels. A radial profile of the remaining image is calculated, which represents the measured $I(\theta)$. A peak finder will mark the local maxima and minima, which corresponds to the green and orange positions in Supplementary Figure 3. As long as the diffraction pattern is clearly visible, maxima and minima will be alternating. As soon as the signal vanishes in noise, however, the peak finding will fail to identify the peak positions correctly and multiple maxima or minima will directly follow each other. At that point any further peaks will be deemed invalid. A straight line will be fitted to all valid peak positions (black dashed in Supplementary Figure 3). The slope of that line is characteristic for the distance between minima and maxima and thus characteristic for the size R of the particle. The slope is used to calculate the estimate for R, that is required as a starting value to fit $I(\theta)$ to the measured $I(\theta)$. Please note that a bias of this estimate is tolerable as the fit will converge towards the measured shape of $I(\theta)$.

In a subsequent step the total number of scattered photons N_{scat} is calculated from the values R and I_0 by integration over the diffraction pattern.

Supplementary Note 5. Calculation of the scattering cross section

Combining the estimated fluence and the parameters R and N_{scat} (calculated from R and I_0, see cluster size fitting model) allows us to calculate the scattering cross section of the Xe nanocluster σ_{sphere} and, subsequently, the scattering cross section of an individual Xe atom σ_{scat}.

Scattering of an individual atom is given by[11]

$$\sigma_{\text{scat}} = \frac{8}{3}\pi r_e^2 |f|^2 \tag{9}$$

A nanocluster consisting of multiple atoms has a much larger scattering which is given by the coherent sum of all contributing atoms (Eq. 30[12]):

$$\sigma_{\text{sphere}} = 2\pi r_e^2 \lambda^2 R^4 \rho^2 |(f_1 + i f_2)|^2 \tag{10}$$

$$= \underbrace{\frac{9}{16\pi}\lambda^2 R\rho}_{=:X_{\text{sphere}}} \cdot N_{\text{atoms}} \cdot \sigma_{\text{scat}} \tag{11}$$

where r_e is the classical electron radius, λ the incident wavelength, R the radius of the nanocluster, ρ the particle density of the nanocluster and f_1 and f_2 are the real and imaginary part of the atomic scattering factor. For simplicity, the terms are rearranged such that X_{sphere} is the factor by which a nanocluster scatters more than the incoherent sum of all its atoms.

Now, the total radiated energy E_{scatt} can be related to the incident fluence F. By definition

$$E_{\text{scat}} = \sigma_{\text{sphere}} \cdot F \tag{12}$$

and the atomic scattering cross section σ_{scat} can be calculated from the measurement via

$$\sigma_{\text{scat}} = \frac{E_{\text{scat}}}{N_{\text{atoms}} X_{\text{sphere}} F} \tag{13}$$

with N_{atoms} as the total number of Xe atoms in the nanocluster. This relation is used in the data analysis to calculate the atomic scattering cross section of Xe. The number of Xe atoms in the cluster N_{atoms} is calculated by multiplying the volume of the cluster and the number density of solid Xenon: $N_{\text{atoms}} = \rho_{\text{Xe}} \frac{4}{3}\pi R^3$ with $\rho_{\text{Xe}} = 1.78 \cdot 10^{28} \frac{\text{atoms}}{m^3}$ [13]

(footnote: In the 5 fs 3d edge run we could in principle estimate the FEL exposure power density for each hit based on the fluorescence yield, but this may be misleading out of consistency considerations as other runs are more dominated by TRs and the fluorescence yield changes substantially)

Supplementary Note 6. Single exposure images

Supplementary Figure 4 shows the detector images corresponding to radial profiles shown in Figure 3 of the main publication, panel d). The images are combined from the rear pnCCD around the beam axis, covering angles from -60 to 60 mrad and the front pnCCD half detector covering angles from 110 mrad and beyond. In the experiment (compare Figure 1 of the main publication panel a: the rear pnCCD was a factor 5.7 further away from the xray focus than the front pnCCD. At all experimental conditions the diffraction image of the Xe cluster clearly extends to the region covered by the front pnCCD. The clusters shown here are of almost identical size. First note, that and the diffraction images taken with 200 fs and XLEAP (<0.5 fs) look almost identical although the 200 fs pulse carried about 60 times more energy! At the lower left image (1500 eV) the front pnCCD was adjusted slightly further from the beam axis resulting in a larger gap between the detectors, visible in Supplementary Figure 4 and Figure 3 of the main publication, panel d).

Further, the high fluorescence levels are clearly visible at the lower left image. It was taken at 1500 eV photon energy, which is why the diffraction ring spacing changed, while the cluster size remained the same. The region marked by the red box was used to evaluate the fluorescence yield, see (see supplement, section Calculation of fluorescence photons per atom). The images from the most intense shots are summarized in additional data.

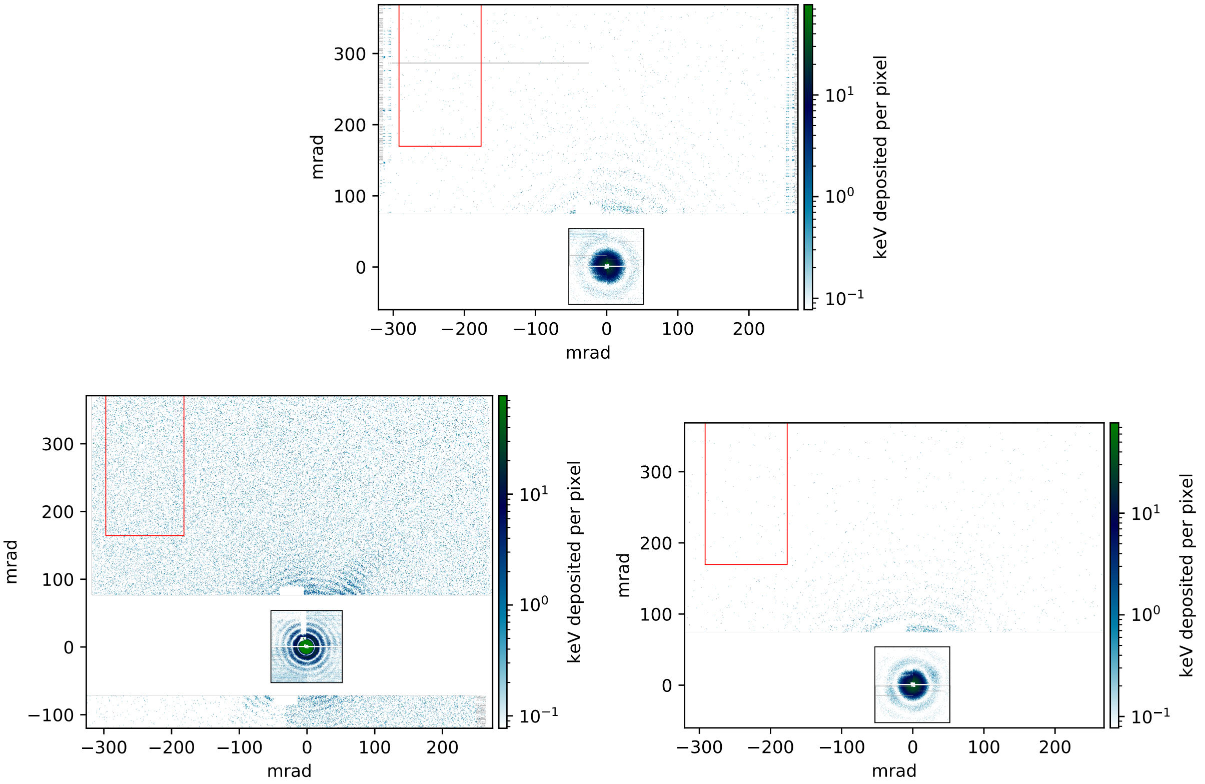

Supplementary Figure 4. Single shot images for the same conditions as shown in figure 1 in the main article: The top image is for the 200 fs pulse, lower left was taken at 5 fs at 1500 eV and lower right image is for XLEAP conditions (<0.5 fs). The image combines the data of the front and the rear pnCCD. The single photons are clearly visible in the images. Please note, that a single photon appears smaller on the rear detector compared to the front detector as each pixel spans a much smaller solid angle. All three particles are of very similar size. The lower left image shows faster diffraction rings because the incident photon energy is 1500 eV compared to ∼700 eV for the other two. The red rectangle indicates the region which has been used to evaluate the fluorescence yield.

Supplementary Note 7. Calculation of fluorescence photons per atom

In contrast to previous studies, which relied on focal averaged Ar gas measurements [10], we exploit the fluorescence yield of single moderately bright images for absolute X-ray exposure fluence calibration. The comparison between the fluorescence yield from the moderate 5 fs measurement (light blue dots) and the M-shell fluorescence yield literature value pointed out as a dashed grey line in Supplementary Figure 5 a) suggests that in this run only up to about 0.3-0.4 X-ray photons/atom were absorbed. TRs play little role if the majority of atoms remain neutral during the FEL exposure and thus, the absolute fluorescence yield is proportional to the exposure FEL fluorescence. Fluorescence photons were detected as single photon hits on the pnCCD detectors. The fluorescence photons per atom (shown in Supplementary Figure 5) are calculated from an outer region of the front pnCCD by summing up all pixels above a threshold corresponding to 125eV. This outer region (marked as a red rectangle in Supplementary Figure 4) is more than 300 mrad from the beam axis, ensuring that even the brightest diffraction patterns have no signal at those large angels and consequently only the fluorescence is measured.

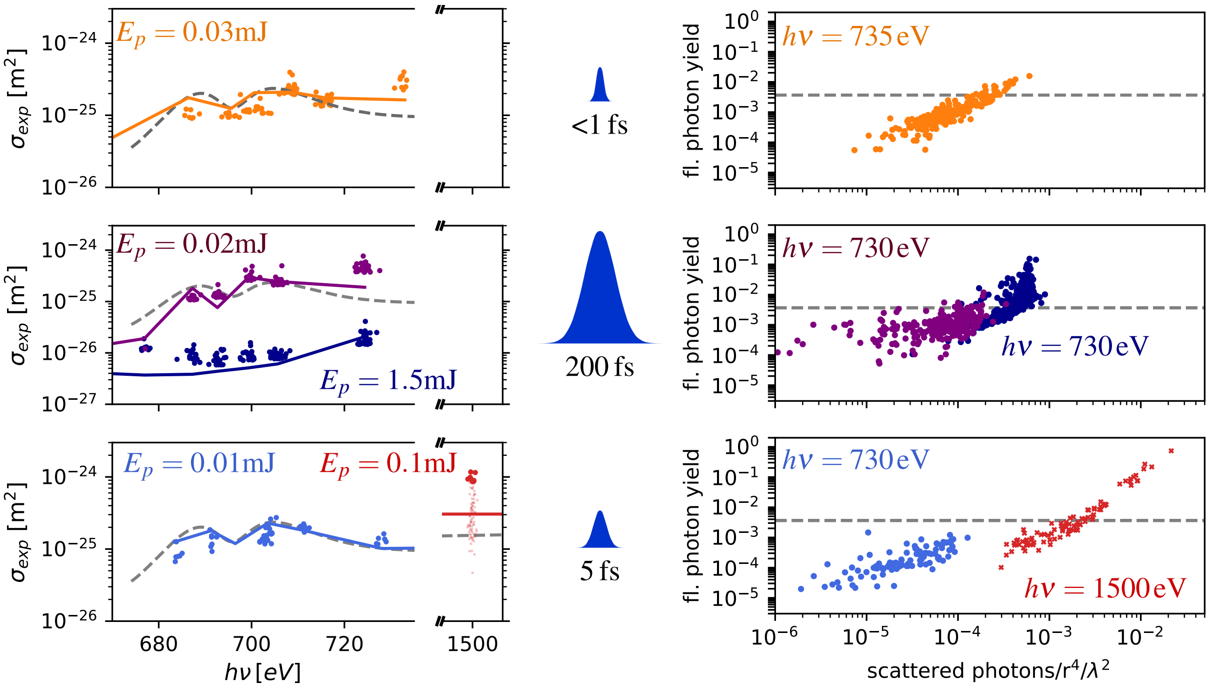

Supplementary Figure 5. The left side depicts the measured σ_{scat}^{exp} in dependence to the incoming X-ray photon energy $h\nu$ for all pulse duration measurements with average pulse energy E_p. The gray dashed line indicates the neutral Xe σ for comparison. Our calculations (solid lines) agree overall well with the experiment. The strongest enhancement can be found at 1500 eV (red) where TR cascades have been observed in the past in Xe atoms [14]. We analyzed all photon energy scan steps with enhanced σ_{exp} as a correlation between scattering and absorption efficiency (represented by fluorescence yield per atom). The measured fluorescence yield per atom is plotted vs the X-ray scattering efficiency for Xe clusters on the right side. The top graph is for the 735 eV with sub-fs pulses measurement, the middle graph is for the 730 eV with 200 fs pulses attenuated and full power measurement, and the lower panel is for the 730 eV with 5-10 fs pulses measurement red and 1500 eV measurement. The scattering and absorption efficiencies are corrected for effects from the cluster radius r and X-ray wavelength λ. The gray dashed line corresponds to tabulated Xe M-shell fluorescence yield 0.00365[15]. Each dot in the graph represents a single cluster observed by a single FEL exposure. The point spread stems from the fact that the nanoparticle diameters 60-150 nm are much smaller than the FEL focus average full width half maximum (FWHM) 1.5 μm. Each randomly injected sample experiences a different exposure FEL fluence inside the FEL focus and only the top few percent of all hits with the highest fluorescence/scattering yields must have been recorded near the FEL focus center with the highest intensities.

The energy deposited on the detector corner is then multiplied by a factor to scale this number to the energy emitted into 4π assuming isotropically emitted fluorescence and a correction for the quantum efficiency of the pnCCDs. It is further divided by the number of atoms in the cluster, which finally yields the "fluorescence photons per atom" as shown in Supplementary Figure 5.

Supplementary Note 8. Signature of transient resonances in the fluorescence yield

A correlation between the σ_{exp} and the number of photons absorbed per atom disentangles the complex interplay between photo-absorption, transient ion states and ultimately, structural damage of the sample. In the right panel of Supplementary Figure 5 the number of scattered photons corrected for the wavelength and particles size is plotted vs the M-shell fluorescence yield per atom (see supplement, section Calculation of fluorescence photons per atom). Both values are extracted from individual snapshots recorded at photon energies above 720 eV. The M-shell fluorescence yield per neutral Xe atom after a single photoabsorption event is only about 0.365 %[15] which is marked as the dotted grey line. Higher fluorescence yield values indicate that multiple photons were absorbed. The number of scattered and absorbed photons depends on F_{ph} which fluctuates mostly because of the random nanoparticle´s position within the FEL focal volume fluence distribution [3, 16–20]. Overall, the number of scattered photons increases with F_{ph} in parallel with the fluorescence yield per atom and the highest yields stem from the FEL focus center.

The correlation between scattering and fluorescence reflects accelerated ionic structure damage during intense 200 fs pulses as well as non-linear increase of the elastic scattering signal on short sub-fs to 10 fs short time scales. First, the images, which are recorded with 200 fs and 1.5 mJ FEL pulses exhibit a saturation behavior where scattering efficiency stagnates with increasing fluorescence (blue dots). This is a clear signature of structural damage of the specimen as demonstrated by previous studies [3, 17, 18, 21, 22]. The scattering efficiency of the entire nanoparticle depends on the ion density which decreases dramatically during the onset of a hydrodynamic expansion. The fluorescence efficiency is less dependent on ion density and individual ions continue to absorb X-rays similarly to the ones inside the intact sample.

Second, the high fluorescence yield of of the brightest images with enhanced σ_{exp} suggests that one or more X-ray photons per atom were absorbed during the FEL exposure. Such high ionization levels within the sample promote the role of transient form factor changes as the probability that X-rays are scattered off an ion with a core-hole rather than a neutral atom increases. This effect is very pronounced in diffraction images recorded with 1500 eV and 5-10 fs FEL pulses where the highest enhancement of σ_{exp} was observed. In the brightest shots, the fluorescence yield increases by two orders of magnitude indicating very high Xe charge states during the FEL exposure. Conversely, σ_{exp} is similar to neutral Xe σ_{scat} in diffraction patterns with low levels of fluorescence recorded with weaker 5 fs pulses at 730 eV (light blue dots, lowerst panel).

Supplementary Note 9. Calibration of incoming X-ray photon energy

An time of flight (tof) spectrum of gaseous Xe was measured, in order to calibrate to an absolute photon energy. Supplementary Figure 6 shows the Xe^{4+} ion yield for different photon energies and compares it to a literature absorption measurement[23]. The double peak structure is a result of the resonance ionization of the Xe $3d_{\frac{3}{2}}$ and $3d_{\frac{5}{2}}$ energy levels. The comparison of the peaks positions with the literature values displays a shift of the incoming XFEL photon energy by only 0.8 eV and was therefore not compensated in the data.

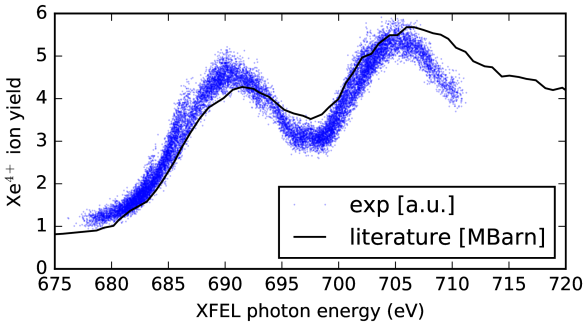

Supplementary Figure 6. Calibration of the incoming photon energy: the literature values (black) are taken from [23]. The peak positions of our measurement and the literature values are only 0.8 eV apart. This difference was therefore not correct in any other data shown in this publication.

References

1. Ho, P. J. *et al.* Atomistic three-dimensional coherent x-ray imaging of nonbiological systems. *Phys. Rev. A* **94,** 063823 (6 Dec. 2016).
2. Ho, P. J. & Knight, C. Large-scale atomistic calculations of clusters in intense x-ray pulses. *J. Phys. B: At. Mol. Opt. Phys.* **50,** 104003 (2017).
3. Ho, P. J. *et al.* The role of transient resonances for ultra-fast imaging of single sucrose nanoclusters. *Nat. Commun.* **11,** 1–9 (2020).
4. Herman, F. & Skillman, S. *Atomic structure calculations* (Englewood Cliffs, NJ:Prentice-Hall, Englewood Cliffs, New Jersey, 1963).
5. Ho, P. J., Bostedt, C., Schorb, S. & Young, L. Theoretical tracking of resonance-enhanced multiple ionization pathways in x-ray free-electron laser pulses. *Phys. Rev. Lett.* **113,** 253001 (DEC 18 2014).
6. Ferguson, K. R. *et al.* The atomic, molecular and optical science instrument at the Linac Coherent Light Source. *J. Synchrot. Rad.* **22,** 492–497 (2015).
7. Strüder, L. *et al.* Large-format, high-speed, X-ray pnCCDs combined with electron and ion imaging spectrometers in a multipurpose chamber for experiments at 4th generation light sources. *Nucl. Instrum. Meth. A* **614,** 483 (2010).
8. Guinier, A. & Fournet, G. *Small-angle scattering of X-rays* (Wiley, 1955).
9. Emma, P. Attosecond X-ray pulses in the LCLS using the slotted foil method. *SLAC pub* (Sept. 2004).
10. Duris, J. *et al.* Tunable isolated attosecond X-ray pulses with gigawatt peak power from a free-electron laser. *Nat. Photon.* **14,** 30–36 (Jan. 2020).
11. Attwood, D. *Soft X-Rays and Extreme Ultraviolet Radiation* (Cambridge University Press, 2012).
12. Howells, M. & Jacobsen, C. Soft X-ray Microscopes and their Biological Applications. *Q. Rev. Biophys.* **28,** 33–130 (Feb. 1995).
13. Klein, M. L. & Venables, J. A. Rare gas solids. *Academic Press London* **2** (1977).
14. Rudek, B. *et al.* Ultra-efficient ionization of heavy atoms by intense X-ray free-electron laser pulses. *Nat. Photon.* **6,** 858–865 (2012).
15. Hubbell, J. H. *et al.* A Review, Bibliography, and Tabulation of K , L , and Higher Atomic Shell X-Ray Fluorescence Yields. *J. Phys. Chem. Ref. Data* **23,** 339–364 (Mar. 1994).
16. Bostedt, C. *et al.* Ultrafast X-Ray Scattering of Xenon Nanoparticles: Imaging Transient States of Matter. *Phys. Rev. Lett.* **108,** 093401 (Feb. 2012).

17. Gorkhover, T. *et al.* Nanoplasma dynamics of single large xenon clusters irradiated with superintense X-ray pulses from the linac coherent light source free-electron laser. *Phys. Rev. Lett.* **108,** 1–5 (2012).

18. Gorkhover, T. *et al.* Femtosecond and nanometre visualization of structural dynamics in superheated nanoparticles. *Nat. Photon.* **10,** 93–97 (Feb. 2016).

19. Hantke, M. F. *et al.* High-throughput imaging of heterogeneous cell organelles with an X-ray laser. *Nat. Photon.* **8,** 943–949 (2014).

20. Ayyer, K. *et al.* 3D diffractive imaging of nanoparticle ensembles using an x-ray laser. *Optica* **8,** 15–23 (Jan. 2021).

21. Flückiger, L. *et al.* Time-resolved x-ray imaging of a laser-induced nanoplasma and its neutral residuals. *New J. Phys.* **18,** 043017 (2016).

22. Peltz, C. *et al.* Few-femtosecond resolved imaging of laser-driven nanoplasma expansion. *New J. Phys.* (2022).

23. Deslattes, R. D. Photoionization of the *M* Shell of Xenon. *Phys. Rev. Lett.* **20,** 483–485 (Mar. 1968).
